# Long‐term outcomes of skull base chordoma treated with high‐dose carbon‐ion radiotherapy

**DOI:** 10.1002/hed.26307

**Published:** 2020-05-30

**Authors:** Masashi Koto, Hiroaki Ikawa, Takashi Kaneko, Yasuhito Hagiwara, Kazuhiko Hayashi, Hiroshi Tsuji

**Affiliations:** ^1^ National Institute of Radiological Sciences, National Institutes for Quantum and Radiological Sciences and Technology Chiba Japan

**Keywords:** carbon‐ion, radiotherapy, long‐term outcome, radiotherapy, rare tumor, skull base chordoma

## Abstract

**Background:**

We evaluated the long‐term efficacy and safety of carbon‐ion radiotherapy (C‐ion RT) for skull base chordoma, a rare neoplasm.

**Methods:**

Thirty‐four patients with skull base chordoma who were treated with C‐ion RT were prospectively enrolled and analyzed retrospectively. C‐ion RT was delivered with 60.8 Gy (relative biological effectiveness [RBE]) in 16 fractions at four fractions per week.

**Results:**

The median follow‐up period was 108 months. The 5‐ and 9‐year local control rates were 76.9% and 69.2%, respectively. The 5‐ and 9‐year overall survival rates were 93.5% and 77.4%, respectively. Regarding grade 3 or more severe late reactions, one patient developed a grade 3 mucosal ulcer, two developed grade 4 ipsilateral optic nerve injuries, and one developed a grade 5 mucosal ulcer at 9 years and 3 months after C‐ion RT.

**Conclusion:**

C‐ion RT with 60.8 Gy (RBE)/16 fractions is a promising treatment option for inoperable skull base chordoma.

## 1 INTRODUCTION

Chordomas are rare neoplasms of the axial skeleton that arise from the remnant of the primitive notochord. About 35% arise from the skull base, where they typically involve the clivus.^1^ Skull base chordomas have a low potential for metastases; therefore, local control is the most important factor for survival.^2^ The standard treatment approach is surgery, and adjuvant radiotherapy is needed for most cases because of locally aggressive growth and the difficulty of radical resection. High‐dose irradiation is recommended despite the adjacent presence of critical organs.^3^ Charged particle therapy using carbon‐ions or protons with the Bragg‐ peak can allow more localized delivery of the radiation dose compared with photon therapy. Several studies have reported promising local control (LC) rates of skull base chordomas treated with charged particle therapy, with 5‐year rates ranging from 69.6% to 75.8%.^4^, ^5^, ^6^, ^7^ However, the slow rate of growth of skull base chordomas may mean that evaluation at 5 years after treatment may not be appropriate. Munzenrider et al reported that, in a series of 169 skull base chordomas treated with photon plus proton therapy, LC at 5 and 10 years was achieved in 73% and 54% of patients, respectively.^6^ Uhl et al reported that, in a series of 155 skull base chordoma treated with carbon‐ion radiotherapy (C‐ion RT), the 5‐ and 10‐year LC rates were 72% and 54%, respectively.^7^

We previously conducted a phase I/II study of C‐ion RT for skull base tumors and recommended a total dose of 60.8 Gy (relative biological effectiveness [RBE]) in 16 fractions over 4 weeks.^8^ After that study, patients with skull base chordomas were prospectively enrolled and treated with an integrated protocol. Thus, in this study, we evaluated the long‐term outcomes of patients with skull base chordoma treated with a dose of 60.8 Gy (RBE) in 16 fractions.

## 2 MATERIALS AND METHODS

A retrospective case review of patients with chordoma of the skull base who were treated with C‐ion RT at 60.8 Gy (RBE) over 16 fractions at our institute between September 2002 and May 2016 was conducted. The following eligibility criteria were employed for patients who received C‐ion RT for skull base chordoma: (a) histologically confirmed chordoma of the skull base, (b) grossly measurable tumor, (c) no distant metastasis, (d) age between 15 and 80 years, (e) an Eastern Cooperative Oncology Group performance status score ≤ 2, (f) medically inoperable tumor or refusal of surgery, and (g) no serious medical or psychological conditions precluding the safe administration of treatment. Patients who previously underwent irradiation for the same lesion were excluded.

A total of 34 consecutive patients were identified and included in the study. The five initial patients were treated in a phase I/II dose escalation study for skull base tumors, while the next 10 were treated as part of a phase II study. After these studies, patients with skull base chordoma were prospectively enrolled and treated using this recommended dose and fractionation protocol. Patients provided informed consent authorizing the use of their personal information for research purposes. This retrospective study was reviewed and approved by the Institutional Ethical Committee on Human Clinical Research (17‐023) and was carried out in accordance with the Declaration of Helsinki. This trial was registered with UMIN‐CTR (http://www.umin.ac.jp/ctr/index-j.htm, identification number UMIN 000029380).

The demographic and tumor characteristics of the 34 patients are shown in Table 1. Carbon‐ion doses are expressed as photon‐equivalent doses in Gy (RBE)^9^ and were defined as the physical dose multiplied by the carbon‐ion RBE. The biological flatness of the spread‐out Bragg peak (SOBP) was normalized using the surviving fraction of human salivary gland tumor cells at the distal SOBP region, resulting in a carbon‐ion RBE of 3.

**TABLE 1:** Patient, tumor, and treatment characteristics

Patient characteristics		Number (%)
Sex	Male/female	18 (53%)/16 (47%)
Age (years)	Median	52
	Range	16‐76
Performance status	0/1/2	12 (35%)/18 (53%)/4 (12%)
Eye symptom	Yes/no	20 (59%)/14 (41%)
Tumor characteristics		
Tumor site	Skull base	34 (100%)
Histology	Chordoma	34 (100%)
Tumor status	Recurrence	7 (20%)
	Residual tumor	22 (65%)
	Naïve tumor	5 (15%)
GTV (cc)	Median	18.7
	Range	1.5‐126.7
Treatment characteristics		
Total dose	60.8 Gy (RBE)	34 (100%)
Fractions	16 fractions (4 fractions a week)	34 (100%)
Treatment period (days)	Median	28
	Range	24‐33
D1cc, Gy (RBE)	Median	58.9
	Range	41.9‐60.4
Beam delivery	Passive	33 (97%)
	Active scanning	1 (3%)

Abbreviations: D1cc, minimum dose received by the maximally irradiated 1 cc of the gross tumor volume; GTV, gross tumor volume; RBE, relative biological effectiveness.

Patients were positioned in customized cradles and immobilized using low‐temperature thermoplastic shells. A set of computed tomography (CT) images of 2 or 2.5‐mm thickness was obtained for treatment planning with the immobilization devices. Magnetic resonance imaging (MRI) was routinely performed for determination of the gross tumor volume (GTV). The clinical target volume (CTV) had margins of 5 to 8 mm added around the GTV and included the entire clivus, inclusive of the original tumor bed. The planning target volume (PTV) had margins of 2 mm added around the CTV. The target reference point dose was defined as the isocenter, and an isodose line representing 90% of the reference point dose encompassed the PTV. The dose limits for critical normal tissues were defined as a maximum point dose of 30 Gy (RBE) for the spinal cord and brain stem and 40 Gy (RBE) for the chiasm and optic nerve. When the ipsilateral optic nerve was located near the GTV, the dose limit for the optic nerve was ignored. The CTV margin of areas close to critical organs, such as the brain, brain stem, and optic nerve, was reduced as necessary. The dose limits of the brain stem, chiasm, and contralateral optic nerve were given priority over PTV coverage. Multiportal irradiation was planned fundamentally to avoid severe normal tissue reactions. A dose limit was not established for the temporal lobe, although irradiation to the temporal lobe was reduced using multiportals and adjustment of CTV margins as much as possible. Three‐dimensional treatment planning was performed using HIPLAN software (National Institute of Radiological Sciences, Chiba, Japan) and Xio‐N (ELEKTA, Stockholm, Sweden and Mitsubishi Electric, Tokyo, Japan). The dose calculation algorithm at our hospital was updated from the HIPLAN to the Xio‐N in 2013. HIPLAN software was used in 29 patients, and Xio‐N was used in 5 patients.

During each treatment session, the patient's position was verified with a computer‐aided online positioning system, and digital orthogonal radiographic images were taken and transferred to the positioning computer. The positioning images were compared with the reference images digitally reconstructed from CT scans. If the difference in positioning was more than 2 mm, the treatment couch was repositioned until the desired position was achieved. The prescribed dose was 60.8 Gy (RBE) in 16 fractions with four fractions per week. Thirty‐three patients were treated with passive irradiation and one with active spot scanning irradiation.

Within the first 5 years, follow‐up examinations were scheduled at intervals of 3 to 6 months and 6 to 12 months thereafter. Radiologic criteria for LC were defined as stable or reduced tumor volume within the PTV on consecutive MRI studies. Any enlargement of the tumor on subsequent radiological studies was considered a local recurrence. Regional control was defined as absence of evidence of tumor recurrence outside the PTV in the skull base and facial bones.

Acute (within 90 days of C‐ion RT completion) adverse reactions were evaluated according to the Radiation Therapy and Oncology Group scoring system. Late (after 90 days) adverse reactions were classified according to the National Cancer Institute Common Terminology Criteria for Adverse Effect version 4.0.

LC, loco‐regional control, overall survival (OS), progression‐free survival (PFS), and distant metastasis rate were assessed from the initiation of C‐ion RT and evaluated using the Kaplan‐Meier method. The log‐rank test was used to compare LC and OS according to clinical factors (sex, age, performance status, tumor status, eye symptom, GTV, and the minimum dose received by the maximally irradiated 1 cc volume of the GTV [D1cc]). Receiver operator characteristic (ROC) curves were used to determine the optimal cutoff value of GTV for prediction of LC. *P*‐values <.05 were considered statistically significant, and all statistical tests were two‐sided. Analyses were performed using SPSS software, version 26 (IBM Corp., Armonk, NY).

## 3 RESULTS

### 3.1 LC and survival

**FIGURE 1:**
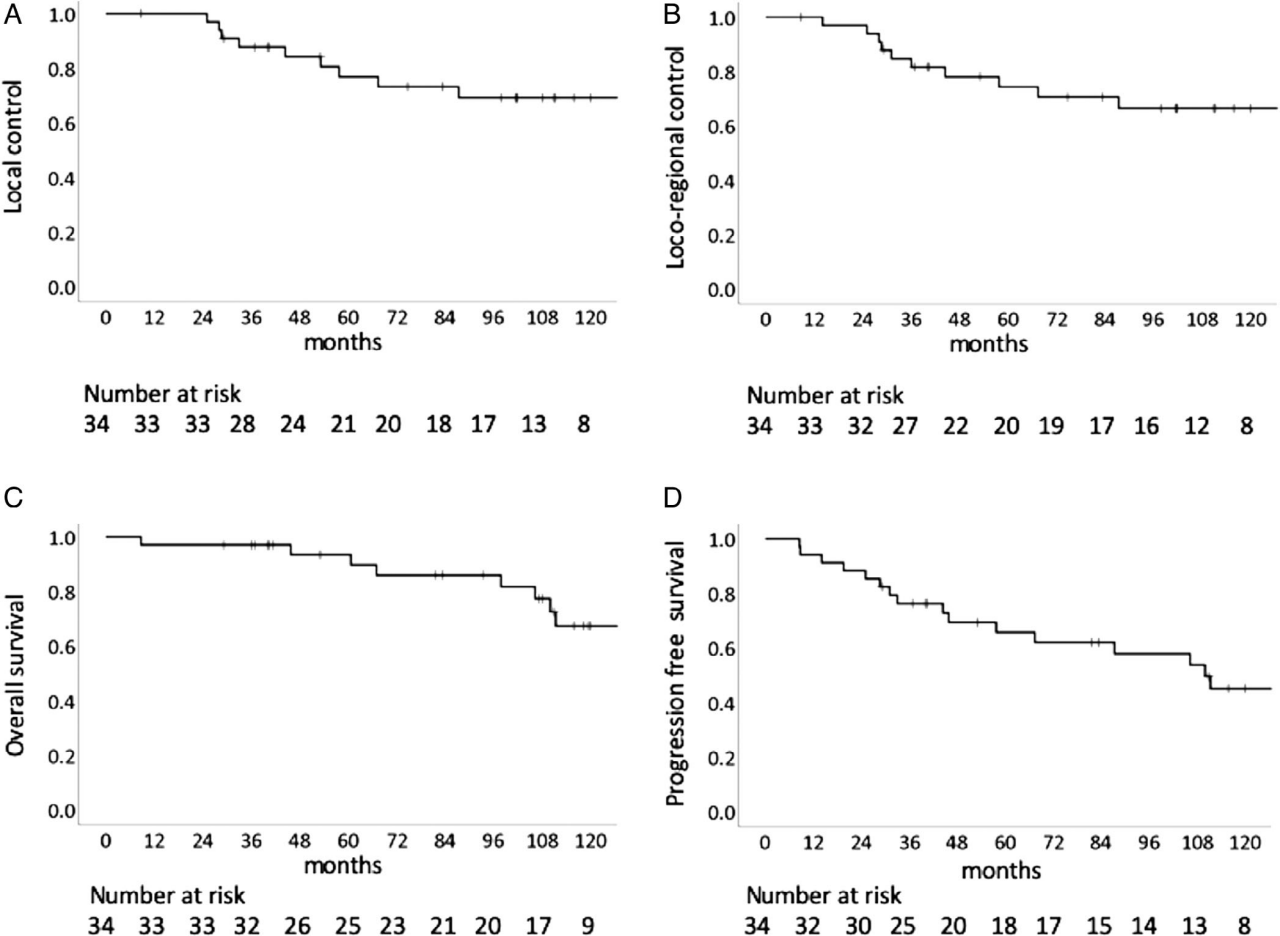
Local control, loco‐regional control, and overall survival. A, Kaplan‐Meier curve of local control (n = 34). B, Kaplan–Meier curve of loco‐regional control (n = 34). C, Kaplan‐Meier curve of overall survival (n = 34). D, Kaplan‐Meier curve of progression free survival (n = 34)

The median observation period was 108 months (range, 9‐175 months), and no patients were lost to follow‐up. The 5‐ and 9‐year LC rates were 76.9% (95% confidence interval [CI]: 61.8%‐92.0%) and 69.2% (95% CI: 52.1%‐86.3%), respectively (Figure 1A). The 5‐ and 9‐year loco‐regional control rates were 74.3% (95% CI: 58.8%‐89.8%) and 66.5% (95% CI: 49.3%‐83.7%), respectively (Figure 1B). Ten patients had local recurrences detected during the follow‐up period, of whom 8 had in‐field recurrences, 1 had marginal recurrence, and 1 had both in‐field and marginal recurrences. Of the 2 patients who developed regional recurrence, 1 each in the skull base and nasal septum, and 1 had local recurrence. Of the 11 patients who developed loco‐regional recurrence, 5 received salvage surgery and 1 underwent re‐irradiation using carbon‐ions. Three patients developed distant metastases in the cerebellum, temporal bone, and cervical spine, respectively. Of these patients, one underwent surgical resection and one received repeated C‐ion RT. The 5‐ and 9‐year estimated distant metastases rates were both 10.1% (95% CI: 0%‐21.1%).

The 5‐ and 9‐year OS rates were 93.5% (95% CI: 84.7%‐100%) and 77.4% (95% CI: 61.3%‐93.5%), respectively (Figure 1C). Of the 8 patients who died, 2 died of their original disease, 1 of bleeding from the nasopharynx, and 5 of other diseases or unknown causes. The 5‐ and 9‐year PFS rates were 65.7% (95% CI: 49.0%‐82.4%) and 53.8% (95% CI: 35.4%‐72.2%), respectively (Figure 1D).

### 3.2 Risk factors for LC and survival

**FIGURE 2:**
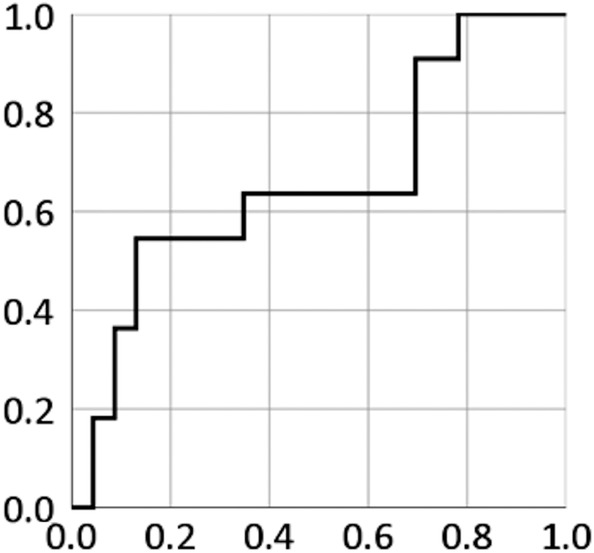
A receiver operator characteristic (ROC) curve for the detection of local control using gross tumor volume. The area under the ROC curve is 0.677 (95% confidence interval: 0.610‐0.744)

Figure 2 shows the ROC curve illustrating the balance between sensitivity and specificity of different values of GTV in predicting LC. The area under the ROC curve was 0.660 (95% CI: 0.453‐0.867). The optimum GTV cutoff value was 34.7 cc, providing a sensitivity of 54.5% and specificity of 87.0%. The results of univariate analyses of risk factors for LC and OS are shown in Table 2. A large GTV was a significant risk factor for poor LC. The 9‐year LC rates of patients with a GTV >34.7 cc and ≤34.7 cc were 26.7% (95% CI: 0%‐57.9%) and 85.7% (95% CI: 70.6%‐100%), respectively. D1cc was not significantly associated with LC; however, two patients with marginal recurrences had lower D1cc compared to those without marginal recurrences. Regarding OS, tumor status, GTV, and D1cc were significant risk factors for OS.

**TABLE 2:** Univariate analysis of local control and overall survival rates

		No. of patients	Local control		Overall survival	
			9‐year (%)	*P*‐value	9‐year (%)	*P*‐value
Sex	Male	18	71.1	.907	72.4	.503
	Female	16	80.8		83.1	
Age (years)	>52	17	94.1	.195	74.3	.401
	≤52	17	59.6		79	
Performance status	0	12	72.9	.158	88.9	.598
	1	18	75.7		78.3	
	2	4	100		50	
Tumor status	Recurrence	7	85.7	.646	85.7	.005
	Residual tumor	22	73.7		87.1	
	Naïve tumor	5	75		n.a.	
Eye symptom	Yes	20	81.6	.097	82.2	.346
	No	14	53		68.6	
GTV (cc)	>34.7	9	26.7	.001	38.1	.022
	≤34.7	25	85.7		90.4	
D1cc (Gy [RBE])	>58.9	15	64	.895	100	.017
	≤58.9	19	71.8		64.4	

Abbreviations: CI, confidence interval; D1cc, minimum dose received by the maximally irradiated 1 cc volume of the GTV; GTV, gross tumor volume; RBE, relative biological effectiveness.

### 3.3 Toxicity

The acute and late adverse reactions that were observed are summarized in Table 3. Two patients (6%) developed ipsilateral blindness due to optic nerve injury subsequent to tumor invasion of ipsilateral orbital apex that necessitated irradiation of the optic nerve with 100% of the prescribed dose. Of the 17 patients that developed brain injuries, 14 (41%) were asymptomatic (grade 1) and 3 (9%) had mild symptoms (grade 2). Two patients (6%) developed small brain stem injuries with no apparent clinical symptoms. One patient with a recurrent skull base chordoma developed fatal bleeding (grade 5) from the nasopharynx at 9 years and 3 months after C‐ion RT. This patient had undergone transsphenoidal surgery 3 years before C‐ion RT, after which they suffered a local recurrence and underwent a second surgery through the transpetrosal approach 1 year prior to C‐ion RT.

**TABLE 3:** Number of acute and late adverse events

	Grade					
	0	1	2	3	4	5
Acute phase						
Dermatitis	20	14	0	0	0	0
Mucositis	15	11	7	1	0	0
Late phase						
Injury of the brain stem	32	2	0	0	0	0
Brain injury	17	14	3	0	0	0
Mucositis	32	0	0	1	0	1
Bone fracture	34	0	0	0	0	0
Optic nerve injury	32	0	0	0	2	0
Hearing impairment	30	0	4	0	0	0
Hypoglossal nerve disorder	33	1	0	0	0	0
Hypopituitarism	33	0	1	0	0	0

## 4 DISCUSSION

Chordomas are locally aggressive and invasive tumors; however, they are usually slow growing. Therefore, a long follow‐up period is needed to determine the precise efficacy of treatments in achieving LC. Munzenrider et al reported that the 10‐year LC rate in 169 patients treated with proton +/− photon therapy was 54%, which was lower than the 73% rate observed at 5 years.^6^ However, the median follow‐up period in their study was 3.4 years. Weber et al also reported in a series of 151 skull base chordoma patients treated with proton therapy that the 7‐year LC rate was 70.9%; however, in their study, the median follow‐up period was 4.2 years.^5^ In a series of 155 patients treated with C‐ion RT, Uhl et al reported 5‐ and 10‐year LC rates of 72% and 54%, respectively, at a median follow‐up of 6.0 years.^7^ In our study, the 9‐year LC rate after high dose C‐ion RT was 69.2% at a median follow‐up period of 9.0 years. To the best of our knowledge, this study presents the longest follow‐up data available for skull base chordomas treated with RT.

While chordomas are radioresistant tumors, they do demonstrate a dose‐response characteristic. Schulz‐Ertner et al demonstrated the dose‐response relationship in chordomas based on the data published in photon and proton studies.^10^ They concluded that more than 75 Gy in a conventional fraction would improve LC. In this study, 60.8 Gy (RBE)/16 fractions, which was equivalent to 88.2 Gy (RBE) in a 2 Gy (RBE) per fraction with a 2‐Gy α/β value if the linear‐quadratic model can be applied to C‐ion RT, was a sufficiently high dose for skull base chordoma. The 5‐year LC rate of 76.9% was slightly lower than what would be expected based on the dose‐response relationship; however, the 9‐year rate of 69.2% is the best outcome reported to data. Nevertheless, tumor size and location may have an impact on LC.

In the treatment of skull base chordomas, improved LC is directly correlated with longer OS because distant metastases are less common. Fung et al reported that, in an analysis of factors associated with OS in 106 chordoma patients, LC was an independent favorable prognostic factor.^2^ In our study, the 5‐ and 9‐year OS rates were 93.5% and 77.4%, respectively.

Regarding risk factors for LC and OS, Fung et al reported that GTV >25 cc was an independent unfavorable prognostic factor for LC.^2^ Weber et al reported the risk factors for LC and OS after proton therapy for skull base chordoma and chondrosarcoma.^5^ They found that GTV >25 cc was also one of the independent risk factors for both LC and OS. Although a large GTV was a risk factor for LC and OS in our study, the cut‐off value based on the ROC analysis was 34.7 cc, which is larger than in previous studies. The high prescribed dose may be one of the reasons underlying this larger tumor size. The 9‐year LC of chordomas with a GTV ≤34.7 cc was 85.7%, while it was only 26.7% in those with a GTV >34.7 cc. An increased dose may be needed to improve LC of large skull base chordomas. In C‐ion RT for head and neck carcinoma and sarcoma, a higher dose has already been used.^11^, ^12^, ^13^ The dose coverage of the PTV or GTV is often inadequate because of dose limits imposed by critical adjacent structures such as the brain stem and optic chiasma. McDonald et al demonstrated that the D1cc of the GTV was a significant prognostic factor for LC and should be ≥74.5 Gy (RBE) in a conventional fraction.^4^ In this study, the D1cc of the GTV was not a significant prognostic factor; however, the two patients that had the lowest D1cc developed marginal recurrences. Surgical therapy for volume reduction and making the space between GTV and organs at risk may help improve LC. Moreover, spot scanning irradiation, which has been already introduced at our facility, potentially allows the dose to be increased while maintaining safety.^14^ In this study, spot scanning irradiation was used for the last patient (3%).

There is no consensus on setting the CTV for skull base chordomas. In this study, a localized irradiation field was used for all patients. Regional recurrences were observed in only two patients (6%), which were salvaged by surgery and re‐irradiation using carbon‐ions, respectively.

Temporal lobe injury is one of the critical complications in high dose proton beam therapy or C‐ion RT for skull base chordomas. In proton therapy for skull base tumors, the incidence of symptomatic temporal lobe injury ranged from 3% to 6%.^2^, ^5^ In C‐ion RT, Schulz‐Ertner et al reported that symptomatic temporal lobe injury was observed in 7.2% of 96 patients.^10^ Grade 2 brain injury was observed in three patients (9%) in this study; however, a high total and fraction dose was used and 33 patients (97%) were treated using passive irradiation. We have previously reported that brain injury after C‐ion RT is correlated with dose volume parameters. Brain volume receiving more than 50 Gy (RBE) in 16 fractions was a significant risk factor for the development of brain injury of ≥grade 2 on MRI findings.^15^ The spot scanning irradiation technique that is currently being used may improve dose distribution and reduce the incidence of brain injury.

Our study had several limitations. First, it was retrospective in nature and involved a small sample size; however, the patients were enrolled prospectively and treated with an integrated treatment protocol. Second, the histological subtype of the tumors was not considered in the analysis of prognostic factors because a sufficient amount of histological samples was not available for all patients. Third, two different dose calculation algorithms were used in this study. The difference of D1cc values between these algorithms was not elucidated.

In conclusion, C‐ion RT using a high dose of 60.8 Gy (RBE)/16 fractions demonstrated a potential to cure skull base chordoma with acceptable toxicity. The novel spot scanning irradiation approach may improve both tumor control and adverse effects.

## CONFLICT OF INTEREST

All authors declare no conflict of interest.
